# Establishment of a New Platform for the Management of Patients After Cardiac Surgery: Descriptive Study

**DOI:** 10.2196/13123

**Published:** 2019-04-01

**Authors:** Zhihui Zhu, Haibo Zhang, Yuehuan Li, Xu Meng, Yuqing Jiao, Ying Qin, Yan Li, Jie Han, Kun Liu, Chongyang Liu

**Affiliations:** 1 Beijing Anzhen Hospital Capital Medical University Beijing China

**Keywords:** follow-up, cardiac surgery, telemedicine

## Abstract

**Background:**

Medical care for the Chinese population has been focused on first-line treatment, but with little follow-up on treated patients. As an important part of clinical work, follow-up evaluations are of great significance for the long-term survival of patients and for clinical and scientific research. However, the overall follow-up rate of discharged patients after surgery has been low for many years because of the limitations of certain follow-up methods and the presence of objective, practical problems.

**Objective:**

This study aimed to construct a new two-way interactive telemedicine follow-up platform to improve the collection of clinical data after cardiac surgery and provide reliable and high-quality follow-up services.

**Methods:**

Computer and network technologies were employed in the context of “Internet +” to develop follow-up databases and software compatible with a mobile network. Postoperative follow-up quality data including the follow-up rate and important postoperative indices were used as standards to evaluate the new follow-up management model after cardiac surgery.

**Results:**

This system has been officially operated for more than 5 years. A total of 5347 patients undergoing cardiac surgery have been enrolled, and the total follow-up rate was 90.22%. In addition, 6349 echocardiographic images, 4717 electrocardiographic images, and 3504 chest radiographic images have been uploaded during follow-up assessments. The international standardized ratio was 20,696 person-times.

**Conclusions:**

This new management follow-up platform can be used to effectively collect clinical data, provide technical support for academic research, extend medical services, and provide more help to patients. It is of great significance for managing patients after cardiac surgery.

## Introduction

As an important part of clinical work, follow-up evaluations are of great significance for the long-term survival of patients and for clinical and scientific research. However, the overall follow-up rate of discharged patients after surgery has been low for many years because of the limitations of certain follow-up methods and the presence of objective, practical problems [1-2]. Collection of large-scale multiagency data in clinical practice is accepted and encouraged for cardiac surgery. From a research perspective, database research can help increase the understanding of cardiac surgery. Among other uses, this research enables the investigation of the effects of disease morbidity and mortality, high-risk groups, differences in health care services, and new equipment and technologies [3].

Medical activities in China tend to focus on “first-line treatment,” and follow-up evaluations of patients are often overlooked. Thus, only the treatment of patients during hospitalization is valued [4]. Traditional patient follow-up methods include outpatient review, text message follow-up, telephone follow-up, WeChat follow-up, and letter follow-up [5-9]. The main factors restricting patient participation in posthospital follow-up include the following: (1) Time effectiveness: Patient enthusiasm in participating in follow-up decreases as the time after discharge increases; (2) Age: Most major diseases affect aging patients, and their follow-up participation rate is low; (3) Communication and cost: Traditional means of contact restrict effective communication between patients and doctors, and most doctors and hospitals require re-examination of patients in a hospital (therefore, the additional costs of transportation, accommodations, meals, and registration are factors); (4) Supervision and guidance: Although patients have basic follow-up training after hospital discharge, they do not participate in the follow-up because they are not supervised; and (5) Geographical restrictions: The follow-up of nonlocal patients in key hospitals is a problem. Therefore, the compliance and follow-up rates of most patients in long-term postoperative follow-up care annually decline, and the patients often drop out.

**Figure 1:**
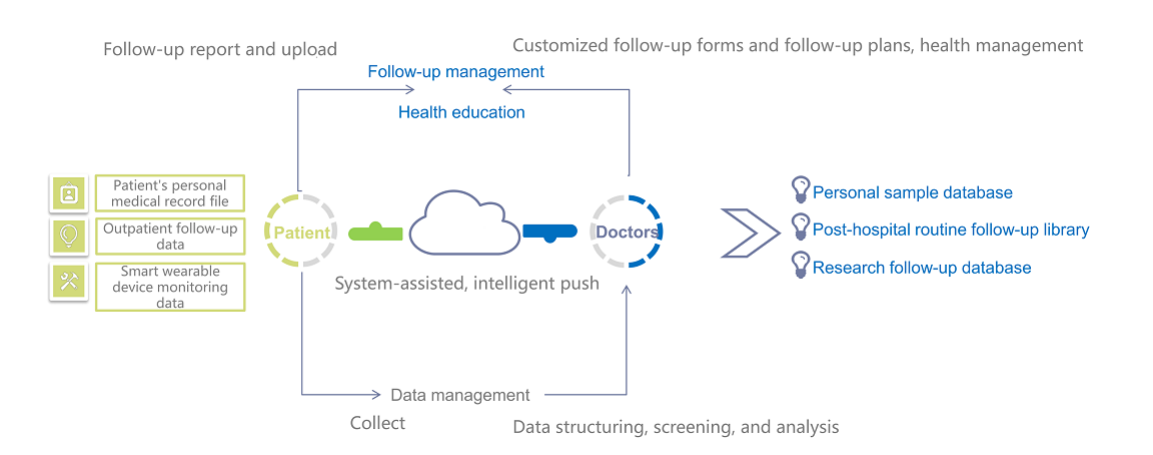
Problems solved by the new follow-up management system.

In order to improve collection of clinical data after cardiac surgery and provide reliable and high-quality follow-up services, we tried to construct a new two-way interactive telemedicine follow-up platform. In 2000, Professor Meng of the Beijing Heart Transplantation and Valvular Surgery Center first applied the informatization management system in clinical care, teaching, and scientific research in China and established the cardiac surgery database. This database was primarily based on the permanent preservation of cardiac surgery information as well as scientific summary and research. Thus far, the database includes more than 20,000 cases of cardiac surgery, and after continuous improvement, the database has operated smoothly. In 2011, based on the original cardiac surgery database, using modern communication tools such as computers and internet technologies, the Beijing Heart Transplantation and Valvular Surgery Center began to work with professional software companies to establish a new two-way interactive telemedicine follow-up system for patients who have been discharged after surgery (see Figure 1 for the problems solved by the new follow-up management system).

Based on the computer network terminals, this telemedicine follow-up system considers the mobile client app as a popular way to coordinate using communication channels such as WeChat, short message service (SMS), and mailbox. The aim of this telemedicine follow-up system is to block abnormal disease processes and improve the quality of life of patients after surgery. This telemedicine follow-up system uses healthy concept inputs, reminders, and recordings as interventions. Through the series of core application functions and intelligent auxiliary methods of this telemedicine follow-up system, the problems encountered by doctors during the follow-up assessments of patients are solved. The follow-up system has been operating officially for more than 5 years, and it has gradually been applied to the cardiac surgery departments of seven hospitals in Beijing. The preparation, operation, maintenance, and follow-up effect of this new follow-up system are summarized below.

## Methods

### Establishment of the Follow-Up Platform

According to the characteristics of thoracic and cardiovascular surgery, the postoperative follow-up requirements of different surgical categories were collected and sorted and the postoperative follow-up index system and follow-up knowledge base were established. Professionals develop follow-up databases and related software that are compatible with mobile networks.

### Development of the Follow-Up System and Standardization of Follow-Up Procedures

#### Patient Source

All patients were discharged from the Beijing Heart Transplantation and Valvular Surgery Center after surgery.

#### Patient Training and Registration

An electronic information registration file and a completed follow-up network registration were established for each patient discharged from the hospital after surgery. The information file includes the patients’ names, genders, dates of birth, contact telephone numbers, hospital numbers, times of admission and discharge, diagnoses, and types of surgery.

All patients and their family members participated in at least three follow-up training sessions before completing the network registration, including watching the mission video, installing the mobile app of the follow-up software, and using the app. Information registration and training for patients discharged from the hospital were completed through the network after confirmation from the follow-up management doctor.

#### Follow-Up Medical Staff Configuration and Workflow

##### Follow-Up Principle

**Figure 2:**
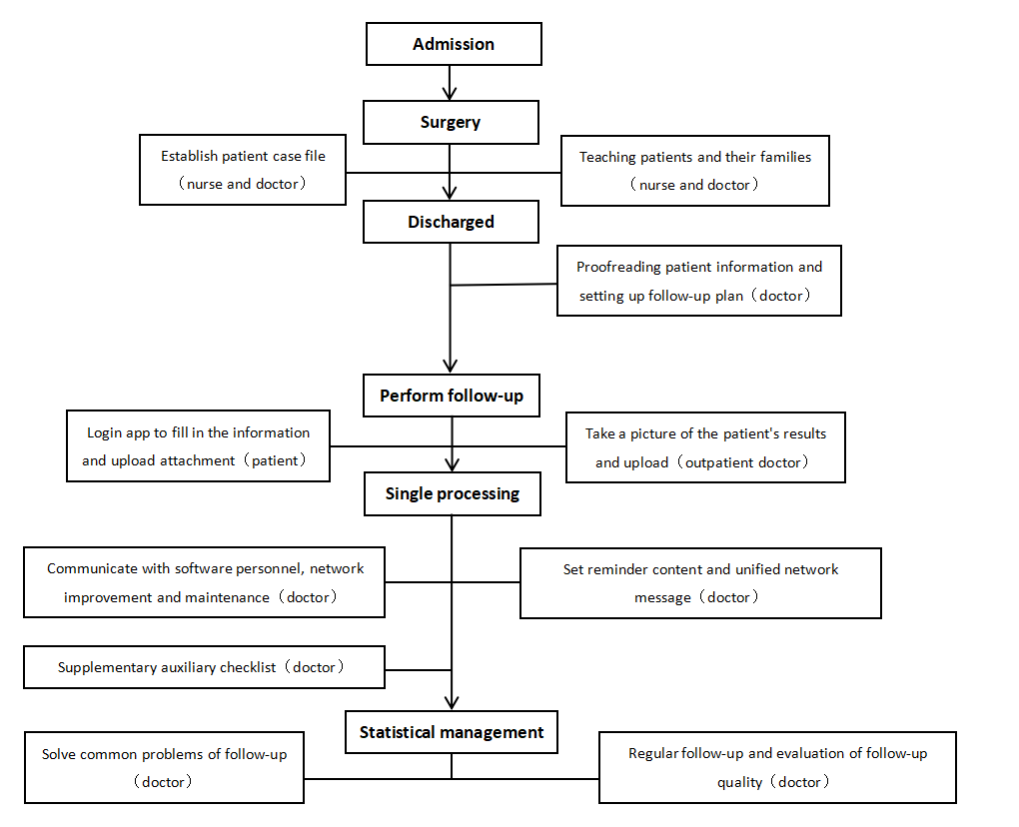
Follow-up workflow of the Beijing Heart Transplantation and Valvular Surgery Center.

The significance of the follow-up platform lies in the entire course of the follow-up assessment, with a review and warning of abnormal indices as the intervention method, which can be distinguished from a general remote follow-up evaluation. In principle, the patient’s report was automatically returned by the follow-up platform. Figure 2 shows the follow-up workflow of the Beijing Heart Transplantation and Valvular Surgery Center.

##### Medical Staff Requirements

Follow-up work was conducted under the leadership of the relevant person from the center, and participation of all medical staff was mandatory. The medical staff of the center were obligated to remind, guide, and strengthen the follow-up care of postoperative patients.

### Statistical Analysis

The follow-up data were exported into an Excel spreadsheet. The patients had to complete at least one report (ie, the reference standard), and follow-up statistics were completed using SPSS 20.0 (IBM Corp, Armonk, NY).

## Results

### Establishment of a New Two-Way Interactive Telemedicine Follow-Up Platform

#### Reminders

Personalized reminders for follow-up questionnaires and the review period were developed based on the diagnosis and surgical approach, which is conducive to enhancing patients’ periodic review and completion of a health self-test.

#### Preliminary Judgment and Comparison Function

For the follow-up indicators and checklists, the system has built-in preliminary judgment and dynamic comparison functions that help patients improve their health awareness and detect problems early.

#### Health Records

The patients’ follow-up questionnaires and the uploaded review data are recorded permanently.

#### Questions and Answers and Training Function

The system regularly summarizes patients’ reports and answers common questions.

#### Clinical Research Assistance

The system can screen, extract, and provide preliminary statistics of the follow-up data.

### Follow-Up List Settings

The follow-up list is determined by the diagnosis and surgical approach. Each patient generally completes two to three follow-up questionnaires, including the regular follow-up, the 36-item Short-Form (SF36) quality of life–assessment scale, and the international standardized ratio (INR) follow-up (for those taking warfarin). According to the actual situation of the center, the routine follow-up list is divided into five categories corresponding to valvular disease/atrial fibrillation (conventional item 1), coronary artery bypass grafting (conventional item 2), valvular disease/atrial fibrillation with coronary artery bypass grafting (conventional item 3), cardiac transplantation (conventional item 4), and congenital heart disease without valvular disease and pericarditis (conventional item 5). Before the patient is discharged from the hospital, the follow-up settings are confirmed via text message.

### Registration of Surgical Patients

By the end of October 2018, 5347 patients receiving cardiac surgery were registered. Of these patients, 4522 were discharged after establishment of the follow-up platform and 825 were discharged before the establishment of the follow-up platform (including 46 deaths). The ages of the patients ranged from 3 to 90 years (average, 51.95 years). A total of 975 patients were aged over 65 years, accounting for 18.3% of the patient population. The male-to-female ratio was 52:48. Further, 3844 patients had heart valve surgery, accounting for 71.89%. There were 259 cases of cardiac transplantation, accounting for 4.84%. The earliest patients were registered was 21 years before the start of this study.

### Follow-Up Details

Some patients do not use the new follow-up method for personal reasons, and we excluded data of these patients. Of the 5234 patients who were enrolled in the follow-up database, 4722 participated in the follow-up, accounting for 90.22%. Of these patients, 2408 were men and 2314 were women. The male-to-female ratio was 1:1, and their ages ranged between 3 and 90 years (average, 51.99 years). Of the 512 patients without follow-up compliance, 267 were men and 245 were women. The male-to-female ratio was 1:1, and their ages ranged between 3 and 85 years (average, 52.95 years). Of the 1021 patients who were discharged from the hospital ≤1 year ago, 893 participated during the follow-up period, and the follow-up rate was 87.46%. Of the 1703 patients who were discharged from the hospital 1-3 years ago, 1553 participated during the follow-up period, and the follow-up rate was 91.19%. Of the 1299 patients who were discharged from the hospital 3-5 years ago, 1193 participated during the follow-up period, and the follow-up rate was 91.84%. Of the 1008 patients who were discharged from the hospital 5-10 years ago, 905 participated during the follow-up period, and the follow-up rate was 89.78%. Of the 203 patients who were discharged from the hospital >10 years ago, 178 participated during the follow-up period, and the follow-up rate was 87.68%.

Of the important follow-up indicators, echocardiography, electrocardiography, chest radiography, blood test/blood biochemistry, INR, and the SF36 scale scores were associated with 6349, 4717, 3504, 1876, 20,696, and 1029 person-times, respectively.

Registration of patients who were discharged before the establishment of the follow-up platform was completed during the follow-up period. Therefore, to reflect the follow-up more objectively, patients who were discharged before the establishment of the follow-up platform were excluded and those who were discharged afterward were analyzed separately. This phase had 4522 registered patients; of these, 3896 participated during the follow-up period, accounting for 86.16%. In addition, 57 patients died after discharge, 19 of whom were involved in the follow-up assessment.

A total of 2497 patients underwent ultrasound examination 4999 times, 1983 patients uploaded electrocardiogram results 3928 times, 1856 patients uploaded chest x-ray results 3021 times, and 898 patients uploaded blood test results 1608 times. Echocardiography, an important indicator in this review, was to be performed during the 3-month follow-up assessment; therefore, patients who were discharged from the hospital less than 3 months ago were excluded, and the echocardiography, electrocardiogram, chest x-ray, and blood test follow-up rates were 75.21% (1732/2303), 59.9% (1074/1793), 54.86% (914/1666), and 25.68% (208/810), respectively.

After mechanical heart valve replacement, patients require long-term use of warfarin, and this group of patients was analyzed separately. Of the registered patients, 1513 underwent mechanical valve replacement, and 1390 completed 16,611 follow-up assessments. The follow-up rate was 91.87%. A total of 9983 patients participated in the INR test; the mean INR was 2.21, and the compliance rate was 57.47%.

### Telephone Interviews With Patients Lost to Follow-Up

After the establishment of the follow-up platform, 1513 discharged patients underwent mechanical valve replacement. The telephone interview results of the 123 patients who did not participate in the follow-up assessment were as follows: 34 patients did not pay attention to postoperative follow-up; 18 patients had mobile phone problems (the primary contact number was a relative’s phone number, not in service, no longer listed, or incorrect) and did not receive reminder text messages; 32 elderly patients did not know how to use the internet, the app, or text messaging; 21 patients directly contacted the doctor in charge; and 15 patients were hospitalized at a local hospital or during the follow-up of the local hospital. In addition, 3 patients had not been examined for INR after discharge.

## Discussion

### Changing the Follow-Up Concept to Improve Compliance

Innovations in information technology have fundamentally changed the way patients perceive time and distance; moreover, they have reshaped the way they interact and connect with others, including how they interact with medicine. As the public becomes more adept with using new technologies in all aspects of daily life, evolving applications in health care will change where and how patients and doctors interact [10]. Over the past four decades, telemedicine has become an increasingly cost-effective alternative to traditional “face-to-face” medicine and has evolved into an integrated technology used in hospitals, clinics, patients’ homes, and many other environments. A growing body of literature has shown that telemedicine electronic media can connect doctors with other doctors, patients with doctors, and even patients with other patients, making clinical treatment and rehabilitation more effective and efficient [11].

Even the most rigorously designed randomized controlled trials are at risk for high rates of loss to follow-up [12]. For many years, improving patient follow-up concepts and compliance has been a major problem in clinical work [13-15]. The biggest innovation of this follow-up system is that patients actively, not passively, complete its report. This innovative concept brings sustainable, self-recognized benefits to patients, and the follow-up system helps patients create profiles. With this follow-up system, patients can describe their condition to the doctor at the next visit. This platform can help patients judge their physical condition and seek medical treatment in time. Past outpatient reviews and remote medical follow-up consultations have focused on individualized diagnosis and treatment; however, it is difficult to address patients’ growing follow-up content in terms of medical risks and costs, if there is no guarantee from the corresponding medical or rehabilitation system. This system can help doctors manage group information of their patients. Doctors can track patients through this system, enriching patients’ data sources. At the same time, this follow-up system can help doctors structure the checklist and build more data sources that can be extracted, analyzed, and applied.

The emergence of electronic health tools provides patients with more opportunities. By learning about specific diseases and receiving regular feedback and frequently enhanced choices, patient engagement can be increased [16]. Solid evidence shows that secondary prevention and cardiac rehabilitation programs reduce mortality among patients with cardiovascular disease after surgery. Internet applications and mobile platforms have broad prospects [13]. The emergence of the follow-up platform helps doctors establish a communication platform for doctors and patients and realize the transmission of health education. The follow-up platform frees some of the doctor’s work during the follow-up. Under limited human and financial conditions, this follow-up platform changed the concept of the follow-up assessment and explored a new balance among patient benefits, costs, and risks.

Through the follow-up training, continuous updating of questions and answers between doctors and patients improved patient health awareness. Individualized follow-up questionnaires, built-in personalized follow-up questionnaires and review periods, system-automated judgments, reminders, and dynamic comparison functions might help patients’ self-test and postoperative adjustment. The health file records basic patient information and postoperative dynamic changes and facilitates future visits and reviews. All system judgments and responses are derived from the built-in procedures of the network, which reduces human hours and time costs and avoids medical risks as much as possible.

### Factors Affecting the Follow-Up Rate

#### Disease Development

Smith et al [17] believed that disease development is an important factor that affects the follow-up rate. Patients with poor prognoses and corresponding complications tend not to participate in follow-up evaluations. However, tracking patients who had been lost to follow-up for more than 10 years, Dexter et al [18] found that these patients were relatively young, were predominately women, and had fewer complications. During our follow-up period, the follow-up rate was 29.8% among the 57 patients who died, which was much lower than the overall follow-up rate. Of the 123 patients lost to follow-up after mechanical valve replacement, 12 (9.8%) had complications; this rate is also significantly higher than that of follow-up in the general population, which suggests that the short-term follow-up rate is associated with disease development. The statistical analysis of all registered patients did not show effects of age or gender on follow-up. In addition, it is difficult to assess the effect of complications on the follow-up period because of the brevity of the follow-up period.

#### Follow-Up Method

The patient follow-up period revealed that speed, the cumbersome nature of computer-based image imports, and internet access location are important factors that restrict the upload of patient checklists. The development of a mobile phone app can solve these problems [19-22]. The new management system provides a mobile phone app for patients, which makes it convenient for patients to upload their own re-examination reports and photos, thereby increasing the portability of the report and making the report more convenient for storage. Furthermore, the system reminds the patient to check and re-examine themselves regularly in the fixed period during the follow-up, which is helpful for patients to receive more active formal postoperative rehabilitation guidance.

Some patients will be familiar with the doctors and will establish channels of communication with doctors, such as telephone, WeChat, and SMS; however, the doctor’s contact information is publicly disclosed, which will affect the doctor’s daily life. The follow-up platform can be used for communication between doctors and patients. Through the patient’s follow-up report, the doctor can control the entire communication process. Through the system’s automatic evaluation of indicators, doctors can choose to communicate only with patients in need. This greatly enhances the follow-up efficiency of doctors. Regarding the mobile aspect, doctors can manage their patients anytime, anywhere.

Outpatient tracking is an important follow-up method after patient discharge [9,23]. Although new technologies and treatment models continue to emerge, the basic ethical responsibilities of doctors have not changed. Medical practice is essentially an ethical activity built on the trust between patients and doctors [24]. Effective communication between these parties is the cornerstone of building trust and providing quality health care [25]. Using this follow-up system, the attending doctor can upload the early outpatient checklist for patients; thus, these patients can go directly to the doctors of the center after the review because the patients have had offline contact with the doctors, thereby establishing a real doctor-patient connection. Furthermore, the patients truly realize that a postoperative follow-up assessment is a key part of the entire rehabilitation based on the center’s satisfactory follow-up training; therefore, they are able to participate in postoperative follow-up care more proactively.

#### Patient Rights and Network Security

Although telemedicine innovation has an enormous potential to benefit patients, it also presents new ethical and security challenges. Of particular concern is that the exchange of patients’ health information and the provision of treatment and training through telemedicine might create new risks to the quality, safety, and continuity of care that could affect the doctor-patient relationship [26-29]. In addition to collection of patient hospitalization information, the establishment of this follow-up platform enables each patient to track the dynamics of the event as much as possible. The follow-up platform is equipped with a follow-up closure function, and the end-point event can be turned off to avoid disturbing the patient. From the patient’s terminal, resetting the password after the first login should help protect patients’ personal information. From the doctor’s terminal, the multirole authority management setting can assign different rights to medical staff, which greatly protects patient privacy and data security.

### Work Plan of the Follow-Up Platform

Because of the brief operation time of the follow-up platform, it is difficult to complete follow-up statistics at many levels. Currently, the activity of the patient is not high enough, and it is necessary to find and optimize derivative services that can be provided to patients to fully mobilize patient participation and compliance. Parker et al [30] analyzed the use of electronics and mobile internet apps among vulnerable groups across 18 telemedicine studies and suggested that encouraging goal setting, providing achievement rewards, strengthening patient responsibility for symptom monitoring, and providing educational guidance and additional support can improve patients’ abilities to manage their diseases. Our future work will improve service design at the patient terminal, optimize the interactive experience, calculate the follow-up rate and follow-up quality of the different follow-up stages, complete the statistics of the follow-up methods and influencing factors, and screen and process the follow-up data for different projects. In addition, we will increase the popularization of science education, provide regular updates, and strengthen interactions with patients. In summary, the follow-up platform will be evaluated and continuously refined from a sustainability perspective.

This follow-up system completes the collection of clinical data after cardiac surgery and provides reliable and quality follow-up services for patients. Based on modern communication technologies, such as computer networks and data storage technology, a new type of follow-up management mode for thoracic and cardiovascular surgery based on wired and wireless network communication was established using the “Internet + medical” operation management mode, supported by the knowledge base of clinical medical diagnosis and treatment. The follow-up management mode provides an open, flexible, and efficient communication platform for doctors, researchers, and discharged patients. Through standardized follow-up behavior and follow-up methods, postoperative health education is strengthened and service is improved. This platform is oriented toward patients, serves clinical research, and provides technical support to comprehensively improve overall medical services and academic research.

## Abbreviations

SMS: short message service
SF36: 36-item Short Form
INR: international standardized ratio
